# Clinical profile, prenatal detection and predictors of outcome of heterotaxy syndromes in Western Australia

**DOI:** 10.1016/j.ijcchd.2023.100472

**Published:** 2023-09-09

**Authors:** Bradley MacDonald, Zoe Vetten, James Ramsay, David Andrews, Deane Yim

**Affiliations:** a Children's Cardiac Centre, Perth Children's Hospital, Perth, Western Australia, Australia; b School of Population and Global Health, University of Western Australia, Perth, Western Australia, Australia; c Department of Paediatric Cardiology, Starship Hospital, Auckland, New Zealand; d Healthy Skin and ARF Prevention, Telethon Kid's Institute, Nedlands, Western Australia, Australia

**Keywords:** Isomerism, Left atrial isomerism, Right atrial isomerism, Heterotaxy, Polysplenia, Asplenia

## Abstract

**Background:**

Heterotaxy syndromes encompass left and right atrial isomerism (LAI and RAI respectively) and are associated with variable cardiac and non-cardiac anomalies which greatly influence outcomes. RAI is usually associated with complex congenital heart disease (CHD), early surgical intervention and increased mortality. LAI is less commonly associated with complex CHD but can be associated with heart block. The objective of this study was to review the clinical features and outcomes of patients with heterotaxy syndromes in Western Australia (WA).

**Methods:**

A retrospective review was performed of live born patients diagnosed with heterotaxy from 2003 to 2022 in a statewide tertiary cardiac service, representing all cases in WA with a view to compare the outcomes between LAI and RAI at our centre.

**Results:**

30 patients (53% male) were diagnosed with heterotaxy; 16 (53%) with LAI and 14 (47%) with RAI. Overall incidence was 0.48 per 10,000 live births over the defined period. RAI patients were significantly more likely to have an antenatal diagnosis (81.8% versus LAI 28.6%, p = 0.03). Overall, 5-year survival was 56% for RAI and 87% for LAI. No deaths occurred after the first 12 months of life with a median follow-up of 65 months (IQR 114.8). RAI was associated with asplenia (91%), atrioventricular septal defect (91%) and a functionally univentricular circulation (71%). LAI was associated with polysplenia (100%) and complete heart block in 3 patients (19%). Surgical pathways included repair of anomalous pulmonary venous return (45%), Blalock Taussig shunt (60%), bidirectional cavopulmonary connection (50%) and Fontan completion (30%).

**Conclusions:**

Patients with RAI suffer high mortality and early surgical intervention, with few making it to Fontan completion. By comparison patients with LAI have less morbidity and mortality. The management of heterotaxy continues to be challenging due to widely associated cardiac and extracardiac manifestations.

## 1 Introduction

Heterotaxy syndromes describe a complex spectrum of congenital cardiac defects characterised by an abnormal arrangement of thoracoabdominal organs across the left-right body axis [1,2]. These syndromes have undergone numerous reiterations to their terminology, including isomerism of the atrial appendages and heterotaxy syndromes with polysplenia or asplenia. Whilst the correct use of nomenclature is outside the scope of this study, we have used the terms of right and left atrial isomerism in our study, in keeping with definitions by Jacobs et al. [3]. Right atrial isomerism (RAI) is typically known as heterotaxy with asplenia, and left atrial isomerism (LAI) describes heterotaxy with polysplenia [1,3]. They are rare, with reported incidence rates of between 1 in 10,000 to 40,000 live births [2,4]. Management of heterotaxy syndromes are challenging because of the wide spectrum of cardiac and non-cardiac anomalies with significant associated morbidity and mortality in early life [5,6]. It remains important to be vigilant of the progress and outcomes of isomerism patients given their complex medical needs.

RAI is commonly associated with complex congenital heart disease (CHD) and a univentricular circulation. Common cardiac lesions in RAI include an atrioventricular septal defect (AVSD), double outlet right ventricle (DORV), pulmonary stenosis or atresia and pulmonary venous anomalies. The presence of obstructed pulmonary veins and a univentricular circulation are associated with poorer outcomes in this group [7,8]. Obstructed total anomalous pulmonary venous return (TAPVR) often precludes staged palliation to Fontan completion [9]. Extracardiac malformations in RAI include gut malrotation that may require prophylactic or corrective surgery [10,11]. In patients that make it to Fontan completion the long-term survival of patients with heterotaxy becomes comparable to other cardiac lesions with similar palliation. This suggests that longer term outcomes may be influenced by the univentricular circulation rather than the underlying diagnosis [12].

Left atrial isomerism present with a wider range of cardiac anomalies and may present later in life due to a lack of earlier symptoms from major CHD. The prevalence of atrioventricular heart block in LAI is higher and may result in fetal demise in utero [13]. They are less likely to be detected antenatally or to require early cardiac surgery than the RAI patients. Outcomes in LAI are highly variable and are related to the type of CHD. Importantly, single ventricle pathology or complete heart block in the prenatal period are associated with a low survival rate and mortality in over 60% in the first year of life [14,15]. Despite improvements with prenatal detection rates, an antenatal diagnosis of heterotaxy discouragingly has had little influence on outcome [4,16,17]. In Western Australia, the addition of the three-vessel view in obstetric ultrasound (in 2013) may have influenced referrals for foetal echocardiogram and diagnosis.

There is a paucity of Australian data for heterotaxy syndromes. Our primary aim was to review the outcomes, including mortality and surgical intervention, between patients with RAI and LAI at our centre. We hypothesised that patients with RAI would have higher mortality and would be more likely to require surgical intervention. Our secondary aims included to review the clinical profile and prenatal detection of patients with heterotaxy syndromes in Western Australia (WA). We also reviewed the influence of updated antenatal screening techniques on the prenatal diagnosis of isomerism.

## 2 Methods

### 2.1 Study design

A retrospective clinical review was performed on all patients diagnosed with heterotaxy at Perth Children's Hospital (known as Princess Margaret Hospital prior to 2018), from January 2003 to December 2022. The hospital represents the only tertiary pediatric Cardiac centre in WA, therefore enabling full statewide capture of heterotaxy cases. Cardiology data bases were searched for relevant patient with terms including ‘isomerism’, ‘heterotaxy’, ‘right atrial isomerism’ and ‘left atrial isomerism’. Cases were included if there was a confirmed diagnosis of right or left isomerism based on echocardiographic data and cross-sectional imaging.

### 2.2 Data sources

Comprehensive data was captured from cardiology clinical (Cardiobase™, Version 8.1.44.10, Derby UK) and echocardiographic (Synapse™ Cardiovascular Client V4.0.4, Fujifilm Medical Systems USA) databases. Details of suspected antenatal diagnoses were, where applicable, collected from a fetal cardiac database (FileMaker Pro v11.0v3). Medical records, where relevant, were used to identify cases and validate the diagnosis of isomerism. Our centre uses established diagnostic criteria for the diagnosis of atrial isomerism on ultrasound [4,18]. Further consensus is reached with review of imaging, including other imaging modalities or surgical findings, within case conferences guided by multiple pediatric cardiologists to consider features making the diagnosis probable (for example the presence of splenic anomalies). LAI was considered probable if meeting criteria or expert consensus was reached on diagnosis due to multiple other factors suggesting diagnosis (polysplenia, biliary atresia, heart block etc.). For RAI, this included asplenia and complex cardiac anatomy. Clinical and demographic information (including ethnicity and postcode), medical and surgical management, outcomes and progress at most recent follow up were reviewed. Outcome measures included type of cardiac surgery or interventions, morbidity or mortality. Predictor variables of interest included cardiac anatomy, anatomical association (i.e. asplenia) as well as specific surgical intervention.

### 2.3 Statistical analysis

Statistical analysis was performed using R version 4.0.3 (R Foundation for Statistical Computing, Vienna, Austria). Descriptive characteristics are presented as counts and percentages where appropriate. Non-normally distributed data are presented as medians and range or interquartile range. Demographic characteristics between LAI and RAI were compared using Pearson's chi-squared test or Fisher's where appropriate; p-values <0.05 were considered significant. Kaplan-Meier curve was used to describe survival probability between LAI and RAI. Cox's proportional hazards model was used to compare survival time. Denominator data of registered births for WA were derived from Australian Bureau of Statistics resources for overall incidence rate [19].

Ethical approval was granted by WA Health Governance, Evidence, Knowledge and Outcomes (GEKO) under registration number #22260.

## 3 Results

### 3.1 Demographics

We identified 30 patients with heterotaxy syndromes born over a 20-year study period. This included 16 (53%) with LAI and 14 (47%) with RAI. Of the patients, 16 (53%) were male with a majority located in the metropolitan area at time of birth (n = 20, 67%). Summary of findings in the cases is detailed in Table 1. No significance difference between proportions was noted between LAI and RAI for biological sex (p = 0.27), ethnicity (p = 0.99) or location (p = 0.095).

**Table 1:** Demographic details of atrial isomerism from 2003 to 2022.

	LAI, N = 16^a^	RAI, N = 14^a^
Current age (months)	100 (35, 178)	32 (4, 90)
Diagnosis		
*Antenatal*	6 (38%)	12 (86%)
*Postnatal*	10 (62%)	2 (14%)
Location		
*Metro*	8 (50%)	12 (86%)
*Regional*	2 (12%)	0 (0%)
*Rural*	6 (38%)	2 (14%)
Sex		
*Female*	9 (56%)	5 (36%)
*Male*	7 (44%)	9 (64%)
Ethnicity		
Aboriginal or Torres Strait Islander	2 (12%)	1 (7.1%)
Other	14 (88%)	13 (93%)
Deceased	2 (12%)	4 (29%)

a Median (IQR); n (%); Range.

Of babies born in WA, this gives us an incidence rate of 0.48 per 10,000 live births over a 19-year period (excluding 2022 due to unpublished ABS data at time of publication). Median age at time of study was 65 months old (IQR 114.8) for the whole cohort; patients with RAI were 32 months (IQR 85.25 months) and LAI were 100 months of age (IQR 143.2 months), with LAI patients being significantly older at time of follow-up (p = 0.032). All deaths occurred within the first year of life.

### 3.2 Anatomical variances

**Fig. 1:**
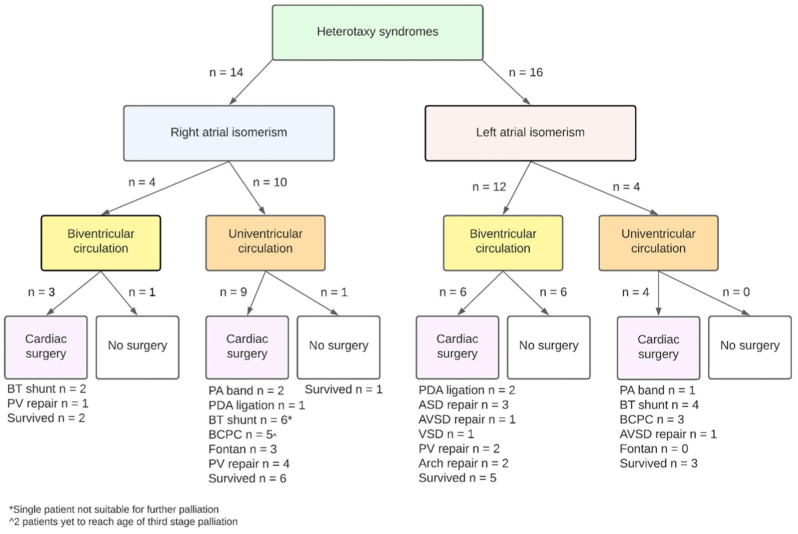
Surgical pathways for univentricular and biventricular circulations in atrial isomerism patients. ASD = atrial septal defect; AVSD = atrioventricular septal defect; BT = Blalock-Taussig Shunt; BCPC = bidirectional cavo-pulmonary shunt; PA = pulmonary artery; PDA = patent ductus arteriosus; PV = pulmonary vein; VSD = ventricular septal defect.

In RAI, the most common findings included atrioventricular septal defect (93%) and TAPVR (86%) (Table 2). Univentricular circulation was seen in 10 patients (71%) with RAI (Fig. 1). There was one patient who had significant atrioventricular valve regurgitationfrom the outset that received palliative care.

**Table 2:** Anatomical associations between LAI and RAI in Western Australia.

Anatomical finding	LAI, N = 16^a^	RAI, N = 14^a^
Atrioventricular septal defect	4 (25%)	13 (93%)
Double outlet right ventricle	2 (12.5%)	9 (64%)
Pulmonary atresia	2 (12.5%)	7 (50%)
Pulmonary stenosis	2 (12.5%)	2 (14%)
Discordant ventriculo-arterial connection	4 (25%)	7 (50%)
Coarctation of the aorta	2 (12.5%)	0 (0%)
Total anomalous pulmonary venous return	4 (25%)	12 (86%)
Obstructed total anomalous pulmonary venous return	2 (12.5%)	5 (36%)
Interrupted inferior vena cava	14 (87%)	0 (0%)
Bilateral superior vena cava	5 (31%)	5 (36%)
Complete heart block	3 (19%)	0 (0%)

a Median (IQR); n (%); Range.

In LAI, 87% of patients had an interrupted inferior vena cava with hemiazygous continuation to the superior vena cava (62%). There were 2 patients with coarctation of the aorta in the LAI group. Common variances are seen in Table 2. Three patients had documented complete heart block. Univentricular circulation was seen in 4 (25%) LAI patients (Fig. 1).

### 3.3 Extracardiac associations

Anatomical associations in RAI included asplenia (n = 13; 93%) and malrotation in 2 patients with Ladd's procedure. There were five patients described as having a midline liver. All LAI patients had polysplenia with 3 (19%) reported as having functional asplenia. There were 2 (12%) patients with LAI who had biliary atresia, with one requiring Kasai procedure, and 7 (44%) with malrotation described with subsequent Ladd's procedure.

### 3.4 Surgical pathways

Surgical pathways remained complex and are detailed in Fig. 1. Most patients (n = 22; 73%) underwent cardiac surgery. A Blalock-Taussig shunt was performed in 8 patients with inadequate antegrade pulmonary blood flow. A bidirectional cavo-pulmonary shunt (BCPC) was achieved in 5 patients with 3 progressing to Fontan circulation. A further 2 patients are expected to reach this stage in the future. In RAI, we found that 21% of our cohort reached Fontan completion, with 14% not yet of age and 14% palliated due to unfavourable haemodynamics and anatomy.

Other surgical pathways for RAI included Ladd's procedure (14%), pulmonary artery banding (14%), PDA ligation (7%) with repair of anomalous pulmonary venous return required (36%). In LAI, this most notably included pacemaker insertion (12.5%), Ladd's procedure (44%), PDA ligation (12.5%), AVSD repair (12.5%), Kasai procedure (6%) and aortic arch repair (12.5%).

There were three RAI (21%) and seven LAI (44%) patients that did not experience any cardiac surgical intervention. In RAI patients, anatomical variance continued to occur with three patients having DORV, two patients with TAPVR and a single patient with pulmonary stenosis. A single patient had a functionally univentricular heart but was being considered for future surgery at the time of data collection.

### 3.5 The influence of antenatal diagnosis

Antenatal diagnosis occurred in 18 patients (60%). In RAI, 12 of the 14 cases (86%) were diagnosed prior to birth. This was significantly less in LAI where only 38% were antenatally diagnosed (p < 0.01). Since 2013 which was the time of implementation of the three-vessel view into routine obstetric scanning in WA, 15 out of the 19 patients (79%) were antenatally diagnosed, including every patient with RAI (n = 9). Antenatal diagnosis was not associated with mortality in the whole cohort (p = 0.18) but was borderline significant in the RAI group (p = 0.06).

### 3.6 Outcomes

**Fig. 2:**
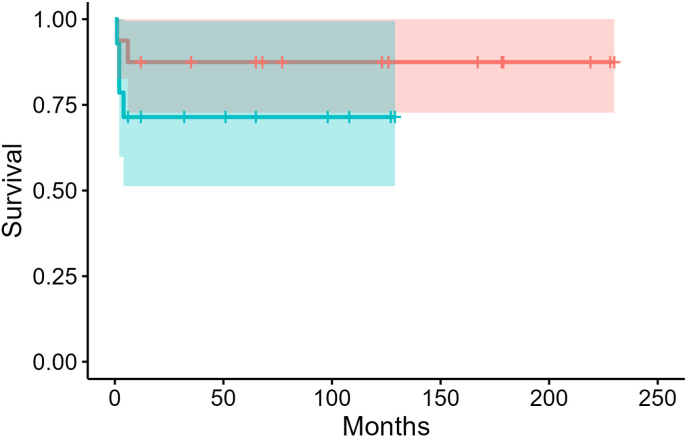
Kaplan-Meier Curve of survival proportion for patients with LAI and RAI showing mortality with 95% confidence intervals as outcome in the first year of life; including end-point of follow up for each patient indicated on each respective line of the graph (red line representing LAI and blue line representing RAI).

Overall survival was 80% at 1-year follow up with no deaths after the first 12 months of life. The 1-year survival was 56% for RAI and for LAI was 87% (hazard ratio 2.51 (0.46–13.7; p = 0.3)). The 5-year survival (n = 16) is 69% for the cohort (Fig. 2). There was no increased mortality seen in patients with AVSD (p = 0.2), DORV (p > 0.9), TAPVR (p = 0.7), pulmonary atresia (p > 0.9) or discordant ventriculo-arterial connection (p = 0.6) (Table 3). There was also no increased mortality associated with BT shunt (p = 0.6), BCPC (p > 0.9) or Fontan (p > 0.9). Obstructed TAPVR was associated with mortality (p = 0.01). Asplenia was not associated with mortality (p = 0.7). Mortality was seen more commonly in patients with patients who had complications such as renal failure (p = 0.03) and requirement of ECMO (p = 0.03). Significant is lost within each subgroup of LAI or RAI specifically (Table 3). A complex array of complications included patients with Blalock-Taussig shunt blockage (n = 2), sepsis (n = 6), endocarditis (n = 2), chylothorax (n = 1) and postoperative bleeds (n = 2) were noted across the groups. In most cases, mortality is likely multifactorial and contributed to complex anatomy, risks of surgery and subsequent complications.

**Table 3:** Comparison of mortality patients and factors contributing to mortality in both LAI and RAI patients.

Variable	LAI, N = 2^a^		RAI, N = 4^a^		Total, N = 6^a^	
	n(%)	p-value	n(%)	p-value	n(%)	p-value
**Demographic**						
Sex (male)	1 (50)	>0.9	3 (75)	>0.9	2 (33)	0.7
Antenatal diagnosis	0 (0)	0.5	2 (50)	0.07	2 (33)	0.2
**Cardiac and other anatomy**						
Functionally univentricular heart	1 (50)	0.4	3 (75)	>0.9	4 (67)	0.7
Atrioventricular septal defect	1 (50)	0.4	4 (100)	>0.9	5 (83)	0.2
Double outlet right ventricle	0 (0)	>0.9	2 (50)	0.6	2 (33)	0.9
Pulmonary atresia	1 (50)	0.4	1 (25)	0.6	2 (33)	>0.9
Pulmonary stenosis	0 (0)	>0.9	0 (0)	>0.9	0 (0)	0.3
Discordant ventriculo-arterial connection	1 (50)	0.4	2 (50)	>0.9	3 (50)	0.6
Coarctation of the aorta	0 (0)	>0.9	0 (0)	NA	0 (0)	>0.9
Total anomolous pulmonary venous return	0 (0)	>0.9	4 (100)	>0.9	4 (67)	0.7
Obstructed total anomalous pulmonary venous return	0 (0)	NA	4 (100)	<0.01	4 (67)	0.01
Complete heart block	0 (0)	>0.9	0 (0)	NA	0 (0)	>0.9
Renal failure	1 (50)	0.13	1 (25)	0.3	2 (33)	0.03
Malrotation	2 (100)	0.13	0 (0)	0.9	2 (33)	0.6
Polysplenia	1 (50)	0.4	0 (0)	NA	1 (17)	0.2
Asplenia	1 (50)	0.4	3 (75)	0.3	4 (67)	0.7
**Intervention/complications**						
Any surgery	1 (50)	0.9	4 (100)	0.5	5 (83)	0.4
Blalock Taussig shunt	1 (50)	0.4	1 (25)	0.6	2 (33)	>0.9
Bidirectional cavo-pulmonary shunt	1 (50)	0.4	0 (0)	0.2	1 (17)	>0.9
Fontan circulation	0 (0)	NA	0 (0)	0.5	0 (0)	>0.9
Atrioventricular septal defect repair	0 (0)	NA	0 (0)	NA	0 (0)	>0.9
Coarctation repair	0 (0)	NA	0 (0)	NA	0 (0)	>0.9
Repair of anomolous venous return	0 (0)	NA	4 (100)	<0.01	4 (67)	0.01
Ladd's procedure	2 (100)	0.2	1 (25)	0.5	3 (50)	0.3
Pacemaker insertion	0 (0)	>0.9	0 (0)	NA	0 (0)	>0.9
Shunt stenosis	0 (0)	>0.9	0 (0)	>0.9	0 (0)	>0.9
Sepsis	1 (50)	0.4	2 (50)	0.07	3 (50)	0.07
Endocarditis	0 (0)	>0.9	1 (25)	0.3	1 (17)	0.4
Extracorporeal membrane oxygenation	0 (0)	NA	2 (50)	0.07	2 (33)	0.03
Chylothorax	0 (0)	NA	0 (0)	>0.9	0 (0)	>0.9
Postoperative bleed	0 (0)	>0.9	0 (0)	>0.9	0 (0)	>0.9

NA = not applicable.

a n (%); Median (IQR); Range.

## 4 Discussion

Heterotaxy syndromes remain a significant challenge for treating clinicians. Our study provides a overview of heterotaxy syndromes in WA, with results similar to larger international cohorts [4,5,9]. We observed that patients with RAI remained at high risk for early mortality and this trend has been similarly reported in surgical series [5,13]. Vodiskar et al. reported an overall mortality of 22.6% with the highest attrition rates seen in the univentricular circulation group, following first stage palliation [20]. Beyond the first year of life, however, we found that mid-term surgical outcomes for surviving patients plateaued and were comparable to other forms of CHD undergoing univentricular palliation, unless obstructed pulmonary veins were present [9].

Not unexpectedly, the biggest challenge in RAI was the development of obstructed pulmonary venous drainage, that was found to be an independent risk factor for mortality. The 5-year survival of RAI was 69%, comparable to other studies [4,9,21]. In addition to pulmonary venous obstruction, patients who developed significant AVVR were often deemed unsuitable for staged operations. One patient who had significant AVVR from the outset was palliated as outcomes for these patients are universally poor [9]. We did not, however, find other anatomical variances beyond obstructed TAPVR to be a risk factor for mortality, although we acknowledge the small cohort may influence the lack of statistical significance.

A wide variation of CHD was witnessed in our LAI group. In contrast to RAI, 75% of CHD in patients with LAI had a biventricular circulation, with corrective surgery required in only half of the cohort. Given the milder forms of CHD, the diagnosis of LAI was later, and the cohort was older than RAI. Congenital heart block occurred in LAI patients; two of which required a pacemaker insertion. Survival rates and mid-term surgical outcomes in the LAI group were superior to the RAI group, as similarly reported in an Australian surgical cohort showing an overall survival of 74% versus 50% in RAI and LAI, respectively, owing to obstructed TAPVR and asplenia [21]. Our study similarly found that a biventricular repair did not confer a survival advantage in RAI, observing 67% survival in both univentricular and biventricular groups undergoing cardiac surgery [21].

We observed an era effect with significant improvements to prenatal detection rates over the past decade to 69% for RAI and 31% for LAI, with no cases to our knowledge diagnosed prior to 2010. This reflects a greater understanding of heterotaxy combined with improved technology and screening since the introduction of the three-vessel view in routine obstetric screening in 2013. Older studies suggest antenatal diagnosis occurs in approximately 50% of patients, with a relatively equal split across LAI and RAI [4]. Our rates of prenatal diagnosis were higher for RAI than LAI, owing to higher rates of complex CHD and anatomical variation in RAI combined with thoracoabdominal situs abnormalities [17]. In addition, in LAI there are known poor prognostic features that can lead to overall lower rates of live births [14]. Similar to previous literature, we did not observe significant differences in outcomes between prenatal and postnatally diagnosed patients with heterotaxy [4].

## 5 Limitations

Our findings were limited by the retrospective nature of the study and small patient numbers given the single-centre series and rarity of the diagnosis. As such, outcome measures may lack statistical power to reach significance. Conversely, however, significant findings that reached statistical significance despite the small sample size, such as obstructed TAPVR as an independent predictor of outcome, further adds weight to this result. Information on widespread non-cardiac anatomical variances associated with isomerism was sometimes unavailable. Additionally, we have not included antenatal cases suffering from fetal demise or termination of pregnancy due to lack of data availability over our period of interest; however, this will be a feature of further research using our fetal echocardiography data. There remain limitations in attributing a single cause of death in this complex group of patients who suffered numerous complications over their lives. Multi-centre collaborative research and national registries would overcome the difficulties of small cohort numbers in rare diseases.

## 6 Conclusion

Management of isomerism remains a significant challenge, with a wide disparity in clinical pathways and outcomes seen with LAI and RAI. RAI is associated with increased mortality in the first year of life, a fact primarily attributed to the presence of obstructed pulmonary venous drainage. Outcomes within right isomerism remain guarded despite improvements in perioperative management and surgical innovation. The management of isomerism continues to be challenging due to widely associated cardiac and extracardiac manifestations. Prenatal diagnosis of heterotaxy has improved considerably in the latter decade, however, did not have a significant impact to longer term outcomes. Further collaborative studies and national databases to study heterotaxy and other rare CHD diseases are needed to increase the cohort numbers, compare clinical experience and expertise and work towards a unified goal of improving longer term outcomes in heterotaxy.

## Disclosures

Not applicable.

## Sources of funding

This research did not receive any specific grant from funding agencies in the public, commercial, or not-for-profit sectors.

## Declaration of competing interest

The authors declare that they have no known competing financial interests or personal relationships that could have appeared to influence the work reported in this paper.
